# Mental health of nursing professionals during the COVID-19 pandemic: a cross-sectional study

**DOI:** 10.11606/s1518-8787.2022056004122

**Published:** 2022-03-14

**Authors:** Luciane Prado Kantorski, Michele Mandagará de Oliveira, Carlos Alberto dos Santos Treichel, Ioannis Bakolis, Poliana Farias Alves, Valéria Cristina Christello Coimbra, Gustavo Pachon Cavada, Lilian Cruz Souto de Oliveira Sperb, Ariane da Cruz Guedes, Milena Hohmann Antonacci, Janaína Quinzen Willrich

**Affiliations:** I Universidade Federal de Pelotas Faculdade de Enfermagem Departamento de Saúde Coletiva Pelotas RS Brasil Universidade Federal de Pelotas. Faculdade de Enfermagem. Departamento de Saúde Coletiva. Pelotas, RS, Brasil; II Universidade de Campinas Departamento de Saúde Coletiva Escola de Ciências Médicas Campinas SP Brasil Universidade de Campinas. Departamento de Saúde Coletiva. Escola de Ciências Médicas. Campinas, SP, Brasil; III Kings College Institute of Psychiatry, Psychology and Neuroscience London United Kingdom Kings College. Institute of Psychiatry, Psychology and Neuroscience. London, United Kingdom

**Keywords:** Nursing, Team, psychology, COVID-19, nursing, Mental Disorders, epidemiology, Mental Health, Occupational Health, Cross-Sectional Studies

## Abstract

**OBJECTIVE:**

To identify the prevalence of and factors associated with: (1) major depressive episodes; (2) minor psychiatric disorders (MPDs); and (3) suicidal ideation among nursing professionals from a municipality in southern Brazil.

**METHODS:**

Using a cross-sectional design, we recruited 890 nursing professionals linked to 50 Primary Care units, 2 walk-in clinics, 2 hospital services, 1 emergency room service, 1 mobile emergency care service, and 1 teleconsultation service, in addition to the municipal epidemiological surveillance service and the vacancy regulation center between June and July 2020. We used the Patient Health Questionnaire-9 and the Self-Reporting Questionnaire to evaluate the studied outcomes. Associations between the outcomes and variables related to sociodemographic profile, work, health conditions, and daily life were explored using Poisson regression models with robust variance estimators.

**RESULTS:**

The observed prevalence of depression, MPDs, and suicidal ideation were 36.6%, 44%, and 7.4%, respectively. MPDs were associated with the assessment of support received by the service as ‘regular’ (PR: 1.48; 95% CI: 1.19–1.85) or ‘poor’ (PR: 1.54; 95% CI: 1.23–1.94), with a reported moderate (PR: 1.63; 95% CI: 1.29–2.07), or heavy (PR: 2.54; 95% CI: 2.05–3.15) workload, and with suspected COVID-19 infection (PR: 1.44; 95% CI: 1.25–1.66). Major depressive episodes were associated with a reported lack of personal protective equipment (PR: 1.20; 95% CI: 1.01–1.42), whereas suicidal ideation was inversely related to per capita income > 3 minimum monthly wages (PR: 0.28; 95% CI: 0.11–0.68), and positively related to the use of psychotropic drugs (PR: 3.14; 95% CI: 1.87–5.26).

**CONCLUSION:**

Our results suggest that nursing professionals’ working conditions are associated with their mental health status. The need to improve working conditions through adequate dimensioning, support and proper biosafety measures is only heightened in the context of the COVID-19 pandemic.

## INTRODUCTION

The COVID-19 pandemic has challenged health systems worldwide^1^ . Nursing professionals constitute approximately half of the global health workforce^2^ , and in the current pandemic, they perform the majority of tasks related to preventing and containing infections. Nursing professionals’ role in caring for COVID-19-infected patients and patients’ family members may have negative consequences to their mental health^3^ .

Increasing COVID-19 cases have coincided with increased workloads, particularly for front-line healthcare professionals. Recognizing potential adverse effects on the mental health of these professionals has instigated research efforts in several countries. Findings, such as the high prevalence of depression, anxiety, sleep disorders, and Minor Psychiatric Disorders (MPDs), stand out^4^ , and some studies point to a higher prevalence of these outcomes among nursing professionals^8 , 9^ . In a comparison of reported hopelessness and anxiety among nurses, doctors, and other health professionals, we observed that nurses’ hopelessness and anxiety levels were significantly higher than the other two groups^8^ .

Daily challenges experienced by nursing professionals are exacerbated by a myriad of psychological stressors during the COVID-19 pandemic, such as high workload, lack of knowledge on the disease, lack of adequate personal protective equipment, and fear of becoming infected and/or infecting loved ones^3 , 4 , 10 , 11^ . In one study, nursing professionals demonstrated a prevalence of 53.5%, 47%, and 38.2% for depression, anxiety, and insomnia, respectively, which were high in comparison to other professionals^6^ .

Despite the high number of recently published studies on health professionals’ mental health in the context of the pandemic, more high-quality investigations are needed. Several published studies on this topic did not employ explicit sample frames and/or had low response rates, which challenges the representativeness of their results^12^ .

In order to increase the evidence base, this study sought to identify the prevalence of, and factors associated with: (1) major depressive episodes; (2) MPDs; and (3) suicidal ideation among nursing professionals from Pelotas, a municipality in the state of Rio Grande do Sul, in southern Brazil. We hypothesized that nursing professionals working in the front lines of the pandemic and those who reported experiencing inadequate working conditions would be more likely to present with adverse mental health outcomes.

## METHODS

### Study Design and Sample

We conducted a cross-sectional study from June to July 2020 with nursing professionals from the municipality of Pelotas (population: 343,132), which serves as a health service and technology reference for 21 other small cities in the surrounding area^13^ . We recruited participants through services aimed at combating the pandemic; namely, 50 Primary Care units, 2 walk-in clinics, 2 hospital services, 1 emergency room service, 1 mobile emergency care service, and 1 teleconsultation service, in addition to the municipal epidemiological surveillance service and the vacancy regulation center. According to the city’s Municipal Health Department, a total of 1,297 nursing professionals worked in these services.

Inclusion criteria for the study were employment as a nursing professional, aging > 18 years, holding a registration with the Regional Nursing Council (COREN), and attending a current employment in a service actively combating the COVID-19 pandemic in the municipality of Pelotas. Exclusion criteria were being on vacation or otherwise absent from work during the data collection period. In addition, a total of 21 professionals who did not provide valid contact information were excluded.

Data collection was conducted using an online, self-administered questionnaire. If they agreed to participate, the nursing professionals were contacted about joining the study and were sent a link to the questionnaire. However, they were required to read and sign an informed consent form before receiving access to begin the questionnaire. This explained the purpose of the study, the participant’s right to decline participation or cease participation at any time, and their right to remain anonymous.

Thus, a total of 944 successful contacts were made among 1,186 eligible professionals; 242 individuals could not be reached. Finally, 54 of those successfully contacted declined to participate, resulting in a 75% response rate (n = 890).

### Measures

The frequency of depressive symptoms in the past 2 weeks was assessed using the 9-question Patient Health Questionnaire (PHQ-9). This instrument scores responses from 0 to 3, and according to a validation study for the general Brazilian population^14^ , a score ≥ 9 provides the highest sensitivity (77.5%; 61.5–89.2) and specificity (86.7%; 83.0–89.9) for screening for major depressive episodes.

The presence of MPDs was assessed using the 20-item Self-Reporting Questionnaire (SRQ-20). The SRQ-20 was also validated for use in Brazil^15^ , and it includes questions on anxiety, depression, and somatic symptoms. All questions are answered with “yes” (1 point) or “no” (0 points), with the highest score being 20 points, and 7 points, identifying the presence or absence of the outcome.

Consistent with previous studies conducted in Brazil^16^ , question 17 of the SRQ-20 instrument was used to screen suicidal ideation. The question asked if the individual “has ever thought about ending their life” in the past 30 days. Suicidal ideation was considered present in participants who answered affirmatively to this question.

### Covariates

Sociodemographic and other COVID-19-related background data were collected using a questionnaire developed by our team. Sociodemographic data consisted of: gender; ethnicity; age; education level; per capita income; type of service; length of service in the nursing field and at the institution; nursing category; workload; information on secondary employment, if applicable; COVID-19-specific training; evaluations of working conditions and support at work; currently perceived burden, and a comparison of burden pre- and post-pandemic period; involvement level with COVID-19 cases; the proportion of the workload involving COVID-19 cases; lack of Personal Protective Equipment (PPE); suspected COVID-19 infection; absence from work due to suspected infection; family members or close friends diagnosed with COVID-19; degree of social distancing/isolation; belonging to the risk group (i.e. those with comorbidities, such as hypertension, diabetes, chronic heart, or respiratory disease, as well as those who had undergone a transplant or were using immunosuppressive drugs); problems with or abuse of alcohol or tobacco; and current use of psychotropic drugs.

### Statistical Analysis

Statistical analyses were conducted using the Stata 16 software program (Stata Corporation, College Station, Texas USA). The prevalence of depression, MPDs, and suicidal ideation were calculated for the full sample and by covariate. Associations of depression, MPDs, and suicidal ideation with the studied covariates were tested using unadjusted and adjusted Poisson regression models with robust variance estimators. The forward stepwise selection was used to select covariates for inclusion in the adjusted analysis following the criterion p ≤ 0.20^17^ .

Potential confounders common to the three outcomes studied (i.e. depression, MPDs, and suicidal ideation) were initially identified. These confounders were gender, age, and per capita income, which composed the first model (model 1) for which each variable was adjusted for each outcome. Next, the confounders for each outcome were identified and a model to adjust the studied variables related to each outcome was created. In Model 2, the dependent variable was major depressive episodes and the covariates entered as potential confounders were gender, age, education, per capita income, evaluation of support at work, burden, lack of PPE, suspected COVID-19 infection, use of tobacco, and use of psychotropic drugs.

The dependent variable in Model 3 was MPD and covariates entered as potential confounders were gender, age, per capita income, evaluation of support at work, burden, suspected COVID-19 infection, use of tobacco, and use of psychotropic drugs. Finally, the dependent variable in Model 4 was suicidal ideation, and covariates included as potential confounders were per capita income, length of service at the institution, workload, secondary employment, evaluation of conditions at work, comparison of burden pre- and post-pandemic, diagnosis of COVID-19 in a family member or close friend, problems with alcohol, and the use of psychotropic drugs.

### Ethical Procedures

The study was reviewed and approved by the Ethics Research Committee in accordance with Brazilian guidelines and standards regulating research involving human beings (Resolution 466/2012) and the Declaration of Helsinki. Ethical principles were upheld as subjects were informed of their right to not participate in the research upon first contact, and a fully informed consent form was signed by all participants. As part of the informed consent process, participants agreed for their anonymized data to be disclosed for scientific purposes. This study adhered to the Guidelines for Strengthening the Reporting of Observational Studies in Epidemiology (STROBE).

## RESULTS

### Characterization of Participants

A total of 944 successful contacts were made among 1,186 eligible professionals; 242 individuals could not be reached because their contact information was either incorrect or no answers were obtained after 10-call attempts on different days, according to a pre-established data collection protocol. In addition, 54 of those successfully contacted declined to participate, resulting in a 75% response rate (n = 890).

Table 1 shows descriptive statistics for the 890 nursing professionals who completed the online questionnaire *.* In the final sample, 319 (35.8%) were registered nurses, 501 (56.3%) were nursing technicians, and 70 (7.9%) were nursing assistants. Most participants were female (n = 755, 84.8%), and the average age was 40.4 years (SD = 8.58). The majority of participants work in a hospital service (n = 577, 64.8%) or in Primary Care (n = 92, 10.3%). Variables with missing data were per capita income (n = 80) and length of service at the institution (n = 5).

**Table 1:** Descriptive statistics for the sample of nursing professionals included in the study (n = 890) (Pelotas-RS, 2020).

	n	%
Gender		
Female	755	84.8
Male	135	15.2
Ethnicity		
White	665	74.7
Brown	122	13.7
Black	103	11.6
Age		
Up to 30	117	13.2
31 to 40	365	41.0
41 to 50	292	32.8
≥ 51	116	13.0
Education level		
High School	330	37.1
Undergraduate	212	23.8
Graduate	348	39.1
Per capita income		
Up to 1 minimum wage	205	25.3
Up to 2 minimum wages	305	37.7
Up to 3 minimum wages	132	16.3
> 3 minimum wages	168	20.7
Type of service		
Primary Care	118	13.3
Outpatient	92	10.3
Emergency	84	9.5
Hospital	577	64.8
Administrative	19	2.1
Length of service in the nursing field		
Up to 5 years	175	19.7
Up to 10 years	221	24.8
Up to 15 years	217	24.4
Up to 20 years	139	15.6
≥ 20 years	138	15.5
Nursing category		
Registered Nurse	319	35.8
Nurse technician	501	56.3
Nursing assistant	70	7.9
Length of service at institution		
Up to 5 years	520	58.8
Up to 10 years	160	18.1
Up to 15 years	71	8.0
≥ 15 years	134	15.1
Workload		
Up to 30h	194	21.8
Up to 36h	473	53.2
36h+	223	25.0
Risk group		
Does not belong to	606	68.1
Does belong to	284	31.9
Involvement with COVID-19 cases		
None	288	32.4
Indirect work (e.g., administrative)	65	7.3
Contact with suspect cases	310	34.8
Contact with confirmed cases	277	25.5
COVID-19 workload proportion		
None	288	32.4
Up to one third	169	19.0
Up to two thirds	166	18.6
> two thirds	267	30.0

### Major Depressive Episodes

Screening indicated a prevalence of 36.6% for major depressive episodes among study participants. Table 2 shows the prevalence of this outcome in association with the study variables of interest and unadjusted and adjusted prevalence ratios.

**Table 2:** Prevalence and unadjusted and adjusted associations between depression and study covariates estimated using Poisson regression models. Data are prevalence ratios (PR) with corresponding 95% confidence intervals (CIs) (n = 890) (Pelotas-RS, 2020).

	n	%	Crude PR (95% CI)	Adjusted^a^ PR (95% CI)	Adjusted^b^ PR (95% CI)
Gender					
Female	755	39.2	1	1	1
Male	135	22.2	0.56 (0.40–0.78)^c^	0.60 (0.42–0.85)^d^	0.66 (0.48–0.91)^d^
Ethnicity					
White	665	31.1	1	1	1
Brown	122	36.9	0.99 (0.77–1.27)	1.03 (0.79–1.34)	0.88 (0.68–1.14)
Black	103	33.0	0.88 (0.66–1.19)	0.87 (0.63–1.19)	0.96 (0.73–1.26)
Age					
Up to 30	117	36.7	1	1	1
31 to 40	365	43.3	1.17 (0.90–1.53)	1.07 (0.82–1.41)	0.89 (0.69–1.16)
41 to 50	292	33.6	0.91 (0.68–1.21)	0.83 (0.62–1.11)	0.69 (0.53–0.91)^d^
≥ 51	116	23.3	0.63 (0.42–0.95)^d^	0.61 (0.40–0.92)^d^	0.55 (0.38–0.80)^d^
Education level					
High School	330	37.6	1	1	1
Undergraduate	212	36.3	0.96 (0.77–1.21)	1.01 (0.79–1.28)	1.04 (0.83–1.30)
Graduate	348	35.9	0.95 (0.78–1.16)	1.20 (0.95–1.50)	1.26 (1.03–1.56)^d^
Per capita income					
Up to 1 minimum wage	205	44.4	1	1	1
Up to 2 minimum wages	305	38.0	0.85 (0.69–1.05)	0.92 (0.75–1.14)	0.79 (0.64–0.96)^d^
Up to 3 minimum wages	132	32.6	0.73 (0.54–0.98)^d^	0.76 (0.57–1.01)	0.66 (0.50–0.86)^d^
> 3 minimum wages	168	29.8	0.67 (0.50–0.88)^d^	0.70 (0.53–0.92)^d^	0.58 (0.44–0.77)^c^
Type of service					
Primary Care	118	33.0	1	1	1
Outpatient	92	34.8	1.05 (0.71–1.53)	0.90 (0.60–1.34)	1.10 (0.78–1.56)
Emergency	84	36.9	1.11 (0.76–1.63)	1.27 (0.87–1.86)	1.11 (0.78–1.57)
Hospital	577	37.9	1.14 (0.87–1.51)	1.12 (0.84–1.49)	1.16 (0.91–1.49)
Administrative	19	26.3	0.79 (0.35–1.76)	0.83 (0.39–1.77)	1.28 (0.61–2.65)
Length of service in the nursing field					
Up to 5 years	175	38.9	1	1	1
Up to 10 years	221	39.4	1.01 (0.79–1.29)	1.02 (0.79–1.33)	0.85 (0.65–1.10)
Up to 15 years	217	37.8	0.97 (0.75–1.25)	1.06 (0.80–1.41)	0.85 (0.65–1.13)
Up to 20 years	139	37.4	0.96 (0.72–1.27)	1.27 (0.92–1.76)	0.93 (0.67–1.29)
≥ 20 years	138	26.8	0.69 (0.49–0.96)	1.13 (0.73–1.75)	0.91 (0.60–1.37)
Nursing category					
Registered Nurse	319	38.6	1	1	1
Nurse technician	501	36.3	0.94 (0.78–1.12)	0.75 (0.61–0.93)^d^	0.77 (0.59–1.00)
Nursing assistant	70	30.0	0.77 (0.52–1.14)	0.95 (0.63–1.44)	1.12 (0.76–1.65)
Length of service at institution					
Up to 5 years	520	38.5	1	1	1
Up to 10 years	160	43.1	1.12 (0.91–1.38)	1.06 (0.85–1.31)	096 (0.79–1.18)
Up to 15 years	71	28.2	0.73 (0.49–1.07)	0.72 (0.49–1.06)	0.75 (0.52–1.08)
≥ 15 years	134	25.4	0.65 (0.47–0.89)^d^	0.90 (0.63–1.29)	0.86 (0.62–1.19)
Workload					
Up to 30h	194	27.3	1	1	1
Up to 36h	473	40.2	1.47 (1.13–1.89)^d^	1.27 (0.97–1.65)	1.03 (0.80–1.32)
36h+	223	37.2	1.36 (1.02–1.81)^d^	1.18 (0.88–1.58)	1.08 (0.83–1.40)
Second job					
No	645	35.7	1	1	1
Yes	245	39.2	1.09 (0.91–1.32)	1.16 (0.95–1.41)	1.06 (0.89–1.27)
COVID-19-specific training					
No	319	39.5	1	1	1
Yes	571	35.0	0.88 (0.74–1.05)	0.91 (0.76–1.09)	0.98 (0.83–1.15)
Evaluation of working conditions					
Good	325	27.1	1	1	1
Regular	409	38.9	1.43 (1.15–1.78)^d^	1.41 (1.13–1.75)^d^	0.91 (0.72–1.15)
Poor	156	54.6	1.87 (1.47–2.36)^c^	1.81 (1.42–2.31)^c^	0.87 (0.64–1.19)
Evaluation of support at work					
Good	263	22.8	1	1	1
Regular	363	38.6	1.69 (1.30–2.18)^c^	1.71 (1.32–2.26)^c^	1.46 (1.14–1.86)^d^
Poor	264	47.7	2.09 (1.61–2.70)^c^	2.05 (1.57–2.66)^c^	1.33 (1.03–1.72)^d^
Burden					
Light	343	18.9	1	1	1
Moderate	262	34.0	1.79 (1.35–2.36)^c^	1.83 (1.38–2.42)^c^	1.67 (1.27–2.18)^c^
Heavy	285	60.3	3.18 (2.50–4.04)^c^	3.26 (2.55–4.16)^c^	2.77 (2.16–3.54)^c^
Current burden compared to that pre- COVID-19					
Same or decreased	337	23.7	1	1	1
Increased	553	44.5	1.87 (1.51–2.31)^c^	1.87 (1.51–2.33)^c^	1.11 (0.89–1.40)
Involvement with COVID-19 cases					
None	288	35.1	1	1	1
Indirect work (e.g., administrative)	65	30.8	0.87 (0.58–1.30)	0.95 (0.64–1.41)	1.07 (0.73–1.55)
Contact with suspect cases	310	35.8	1.02 (0.82–1.26)	0.97 (0.78–1.21)	0.84 (0.68–1.03)
Contact with confirmed cases	277	41.4	1.18 (0.94–1.47)	1.18 (0.94–1.49)	1.03 (0.83–1.29)
COVID-19 workload proportion					
None	288	35.1	1	1	1
Up to one third	169	30.8	0.87 (0.66–1.15)	0.84 (0.63–1.12)	0.77 (0.59–0.99)^d^
Up to two thirds	166	35.5	1.01 (0.78–1.31)	0.98 (0.75–1.27)	0.94 (0.73–1.19)
> two thirds	267	42.7	1.21 (0.98–1.50)	1.23 (0.99–1.53)	1.01 (0.82–1.25)
Lack of Personal Protective Equipment					
No	508	30.7	1	1	1
Yes	382	44.5	1.44 (1.21–1.72)^c^	1.42 (1.19–1.69)^c^	1.20 (1.01–1.42)^d^
Suspected COVID-19 infection					
No	574	30.5	1	1	1
Yes	316	47.8	1.56 (1.32–1.85)^c^	1.57 (1.32–1.87)^c^	1.29 (1.09–1.53)^d^
Absence from work due to suspected infection					
No	746	34.6	1	1	1
Yes	144	47.2	1.36 (1.11–1.66)^d^	1.35 (1.10–1.66)^d^	1.06 (0.86–1.32)
Family or close friend with COVID-19					
No	616	34.1	1	1	1
Yes	274	42.3	1.24 (1.04–1.48)^d^	1.20 (1.00–1.44)^d^	1.01 (0.85–1.20)
Degree of social distancing					
Light	227	29.5	1	1	1
Moderate	568	41.2	1.39 (1.11–1.74)^d^	1.35 (1.07–1.71)^d^	1.24 (0.99–1.54)
Intense	95	26.3	0.89 (0.60–1.31)	0.88 (0.59–1.31)	0.88 (0.61–1.26)
Risk group					
No	606	32.7	1	1	1
Yes	284	45.1	1.37 (1.16–1.63)^c^	1.46 (1.22–1.74)^c^	1.18 (0.99–1.40)
Problems with/abuse of alcohol					
No	824	34.9	1	1	1
Yes	66	57.6	1.64 (1.31–2.06)^c^	1.62 (1.28–2.05)^c^	1.18 (0.94–1.47)
Tobacco use					
No	748	34.4	1	1	1
Yes	142	48.6	1.41 (1.16–1.72)^c^	1.45 (1.18–1.77)^c^	1.32 (1.07–1.62)^d^
Current use of psychotropic drugs					
No	703	31.9	1	1	1
Yes	187	54.5	1.71 (1.44–2.02)^c^	1.74 (1.46–2.07)^c^	1.59 (1.34–1.89)^c^

**TOTAL**	**880**	**36.6**			

^a^ Adjusted for: gender; age; and per capita income.

^b^ Adjusted for: gender; age; education level; per capita income; work support evaluation; burden; lack of Personal Protective Equipment; suspected contagion; tobacco problems; and current use of psychotropic drugs.

^c^ p-value < 0.001

^d^ p-value < 0.05

We observed evidence of an inverse association between screening positive for major depressive episodes and being male (PR: 0.66; 95% CI: 0.48–0.91), aging between 41 and 50 (PR: 0.69; 95% CI: 0.53–0.91), or above 51 years of age (PR: 0.55; 95% CI: 0.38–0.80). A lower prevalence ratio was associated with higher income, for example, professionals whose per capita income was greater than three minimum monthly wage (PR: 0.58 (95% CI: 0.44–0.77).

Having up to one-third of the workload devoted to caring for patients with COVID-19 was inversely associated with major depressive episodes (PR: 0.77; 95% CI: 0.59–0.99). However, no associations were observed in cases where nursing professionals reported dedicating more than one-third of their workload to pandemic-related duties. At the same time, we found evidence for associations among major depressive episodes; having a graduate-level education (PR: 1.26; 95% CI: 1.03–1.56); assessing support from the service as regular (PR: 1.46; 95% CI: 1.14–1.86), or poor (PR: 1.33; 95% CI: 1.03–1.72); reporting a moderate (PR: 1.67; 95% CI: 1.27–2.18) or heavy (PR: 2.77; 95% CI: 2.16–3.54) burden at work; lack of PPE (PR: 1.20; 95% CI: 1.01–1.42); suspected COVID-19 infection (PR: 1.29; 95% CI: 1.09–1.53); use of tobacco (PR: 1.32; 95% CI: 1.07–1.62); and current use of psychotropic drugs (PR: 1.59; 95% CI: 1.34–1.89).

In the model where the adjustment was performed for all potential confounders related to the outcome, the variable related to the evaluation of working conditions showed an association direction contrary to that observed in the crude analysis and in the first model. This appeared to be due to the influence of the variable assessing support received at work. Following the removal of the support variable, however, the working conditions variable was not associated with the outcome, adjusting for other confounders.

### Minor Psychiatric Disorders (MPDs)

The prevalence of MPDs in the sample was 43.9% (n = 391). Table 3 shows the prevalence of this outcome in association with the study variables of interest and unadjusted and adjusted prevalence ratios.

**Table 3:** Prevalence and unadjusted and adjusted associations between minor psychiatric disorders and studied covariates estimated using Poisson regression models. Data are prevalence ratios (PR) and 95% confidence intervals (CIs) (n = 890) (Pelotas-RS, 2020).

	n	%	Crude PR (95% CI)	Adjusted^a^ PR (95% CI)	Adjusted^b^ PR (95% CI)
Gender					
Female	755	47.0	1	1	1
Male	135	26.7	0.56 (0.42–0.75)^c^	0.54 (0.39–0.75)^c^	0.57 (0.42–0.77)^c^
Ethnicity					
White	665	43.3	1	1	1
Brown	122	50.8	1.17 (0.96–1.42)	1.22 (1.00–1.50)^d^	1.03 (0.85–1.24)
Black	103	39.8	0.91 (0.71–1.18)	0.91 (0.68–1.20)	0.94 (0.74–1.19)
Age					
Up to 30	117	47.9	1	1	1
31 to 40	365	52.0	1.08 (0.87–1.34)	1.07 (0.85–1.34)	0.93 (0.75–1.14)
41 to 50	292	39.0	0.81 (0.64–1.03)	0.80 (0.62–1.03)	0.71 (0.56–0.90)^d^
≥ 51	116	26.7	0.55 (0.39–0.79)^d^	0.56 (0.39–0.82)^d^	0.56 (0.40–0.78)^d^
Education level					
High School	330	44.2	1	1	1
Undergraduate	212	43.9	0.99 (0.81–1.20)	1.02 (0.83–1.25)	1.05 (0.87–1.26)
Graduate	348	43.7	0.98 (0.83–1.17)	1.07 (0.88–1.31)	1.12 (0.93–1.34)
Per capita income					
Up to 1 minimum wage	205	49.8	1	1	1
Up to 2 minimum wages	305	42.9	0.86 (0.71–1.04)	0.94 (0.78–1.13)	0.85 (0.72–1.01)
Up to 3 minimum wages	132	40.1	0.80 (0.62–1.03)	0.84 (0.66–1.07)	0.81 (0.66–0.99)^d^
> 3 minimum wages	168	39.3	0.78 (0.62–0.99)	0.83 (0.66–1.05)	0.79 (0.64–0.98)^d^
Type of service					
Primary Care	118	43.2	1	1	1
Outpatient	92	40.2	0.93 (0.67–1.28)	0.80 (0.57–1.12)	0.91 (0.68–1.22)
Emergency	84	32.1	0.74 (0.51–1.08)	0.80 (0.55–1.18)	0.68 (0.47–0.97)^d^
Hospital	577	46.8	1.08 (0.86–1.35)	1.00 (0.80–1.26)	1.00 (0.82–1.23)
Administrative	19	31.6	0.73 (0.36–1.46)	0.74 (0.38–1.43)	1.14 (0.60–2.16)
Length of service in the nursing field					
Up to 5 years	175	47.4	1	1	1
Up to 10 years	221	48.9	1.03 (0.83–1.26)	1.06 (0.84–1.33)	0.96 (0.78–1.18)
Up to 15 years	217	45.2	0.95 (0.76–1.17)	1.07 (0.84–1.37)	0.94 (0.76–1.17)
Up to 20 years	139	41.7	0.87 (0.68–1.13)	1.17 (0.88–1.56)	0.99 (0.76–1.28)
≥ 20 years	138	31.9	0.67 (0.50–0.89)^d^	1.06 (0.72–1.56)	1.00 (0.71–1.42)
Nursing category					
Registered Nurse	319	46.1	1	1	1
Nurse technician	501	43.9	0.95 (0.81–1.11)	0.87 (0.72–1.06)	0.86 (0.72–1.02)
Nursing assistant	70	34.3	0.74 (0.52–1.05)	0.96 (0.64–1.42)	1.11 (0.77–1.59)
Length of service at institution					
Up to 5 years	520	46.1	1	1	1
Up to 10 years	160	50.6	1.09 (0.91–1.31)	1.03 (0.85–1.25)	0.97 (0.81–1.16)
Up to 15 years	71	35.2	0.76 (0.54–1.06)	0.79 (0.57–1.09)	0.80 (0.60–1.06)
≥ 15 years	134	31.3	0.67 (0.51–0.88)^d^	0.97 (0.71–1.33)	0.98 (0.74–1.29)
Workload					
Up to 30h	194	35.0	1	1	1
Up to 36h	473	48.2	1.37 (1.11–1.70)^d^	1.21 (0.96–1.52)	0.95 (0.78–1.17)
36h+	223	42.6	1.21 (0.95–1.55)	1.10 (0.85–1.42)	0.97 (0.78–1.21)
Second job					
No	645	45.1	1	1	1
Yes	245	40.8	0.90 (0.76–1.07)	0.96 (0.80–1.15)	0.87 (0.74–1.02)
COVID-19-specific training					
No	319	43.9	1	1	1
Yes	571	44.0	1.00 (0.85–1.16)	1.01 (0.86–1.18)	1.10 (0.95–1.27)
Evaluation of working conditions					
Good	325	29.8	1	1	1
Regular	409	47.2	1.58 (1.29–1.92)^c^	1.59 (1.30–1.94)^c^	1.13 (0.90–1.41)
Poor	156	64.7	2.16 (1.77–2.65)^c^	2.19 (1.77–1.94)^c^	1.17 (0.89–1.53)
Evaluation of support at work					
Good	263	26.2	1	1	1
Regular	363	44.9	1.71 (1.35–2.15)^c^	1.74 (1.37–2.20)^c^	1.48 (1.19–1.85)^c^
Poor	264	60.2	2.29 (1.83–2.87)^c^	2.27 (1.79–2.86)^c^	1.54 (1.23–1.94)^c^
Burden					
Light	343	23.6	1	1	1
Moderate	262	42.0	1.77 (1.40–2.25)^c^	1.79 (1.40–2.29)^c^	1.63 (1.29–2.07)^c^
Heavy	285	70.2	2.97 (2.42–3.64)^c^	3.02 (2.44–3.74)^c^	2.54 (2.05–3.15)^c^
Current burden compared to that pre- COVID-19					
Same or decreased	337	28.8	1	1	1
Increased	553	53.2	1.84 (1.53–2.22)^c^	1.87 (1.54–2.27)^c^	1.16 (0.94–1.42)
Involvement with COVID-19 cases					
None	288	41.3	1	1	1
Indirect work (e.g., administrative)	65	44.6	1.07 (0.79–1.46)	1.07 (0.79–1.46)	1.22 (0.92–1.61)
Contact with suspect cases	310	42.9	1.03 (0.86–1.25)	0.99 (0.81–1.19)	0.88 (0.74–1.05)
Contact with confirmed cases	277	48.5	1.17 (0.96–1.42)	1.13 (0.92–1.39)	1.01 (0.83–1.21)
COVID-19 workload proportion					
None	288	41.3	1	1	1
Up to one third	169	42.6	1.03 (0.82–1.28)	0.99 (0.79–1.24)	0.94 (0.77–1.15)
Up to two thirds	166	42.7	1.03 (0.82–1.29)	0.97 (0.77–1.23)	0.95 (0.77–1.18)
> two thirds	267	48.3	1.16 (0.97–1.40)	1.14 (0.94–1.38)	0.96 (0.81–1.15)
Lack of Personal Protective Equipment					
No	508	37.4	1	1	1
Yes	382	52.6	1.40 (1.21–1.63)^c^	1.39 (1.19–1.62)^c^	1.13 (0.98–1.31)
Suspected COVID-19 infection					
No	574	35.4	1	1	1
Yes	316	59.5	1.68 (1.45–1.94)^c^	1.68 (1.45–1.96)^c^	1.44 (1.25–1.66)^c^
Absence from work due to suspected infection					
No	746	41.4	1	1	1
Yes	144	56.9	1.37 (1.16–1.62)^c^	1.35 (1.14–1.61)^c^	0.98 (0.82–1.17)
Family or close friend with COVID-19					
No	616	39.9	1	1	1
Yes	274	52.9	1.32 (1.14–1.53)^c^	1.28 (1.10–1.50)^d^	1.07 (0.93–1.24)
Degree of social distancing					
Light	227	36.6	1	1	1
Moderate	568	47.2	1.29 (1.06–1.56)^d^	1.30 (1.05–1.59)^d^	1.19 (0.99–1.44)
Intense	95	42.1	1.15 (0.86–1.54)	1.19 (0.88–1.61)	1.14 (0.87–1.50)
Risk group					
No	606	40.8	1	1	1
Yes	284	50.7	1.24 (1.07–1.44)^d^	1.40 (1.20–1.63)^c^	1.14 (0.99–1.32)
Problems with/abuse of alcohol					
No	824	42.3	1	1	1
Yes	66	63.6	1.50 (1.23–1.83)^c^	1.51 (1.23–1.84)^c^	1.14 (0.94–1.38)
Tobacco use					
No	748	41.3	1	1	1
Yes	142	57.7	1.39 (1.18–1.64)^c^	1.39 (1.17–1.65)^c^	1.22 (1.03–1.45)^d^
Current use of psychotropic drugs					
No	703	40.3	1	1	1
Yes	187	57.7	1.43 (1.23–1.67)^c^	1.46 (1.25–1.71)^c^	1.34 (1.15–1.57)^c^

**TOTAL**	**880**	**43.9**			

^a^ Adjusted for: gender; age; and per capita income.

^b^ Adjusted for: gender; age; per capita income; work support evaluation; burden; suspected contagion; tobacco problems; and current use of psychotropic drugs.

^c^ p-value < 0.001

^d^ p-value < 0.05

Similar to the major depressive episode outcome, we observed an inverse association between screening for MPDs and the male gender (PR: 0.57; 95% CI: 0.42–0.77), aging between 41 and 50 years (PR: 0.71; 95% CI: 0.56–0.90), or above 51 years of age (PR: 0.56; 95% CI: 0.40–0.78). A lower prevalence ratio of MPD was also found for professionals working in emergency services (PR: 0.68; 95% CI: 0.47–0.97), and for those whose per capita income was up to three minimum monthly (PR: 0.81; 95% CI: 0.66–0.99), or greater than three minimum monthly wages (PR: 0.79; 95% CI: 0.64–0.98).

We found evidence for positive associations of MPD with the following variables: assessment of support received by the service as regular (PR: 1.48; 95% CI: 1.19–1.85) or poor (PR: 1.54; 95% CI: 1.23–1.94); reported moderate (PR: 1.63; 95% CI: 1.29-2.07) or heavy (PR: 2.54; 95% CI: 2.05–3.15) burden at work; suspected COVID-19 infection (PR: 1.44; 95% CI: 1.25–1.66); current use of psychotropic drugs (PR: 1.34; 95% CI: 1.15–1.57); and tobacco use (PR: 1.22; 95% CI: 1.03–1.45).

### Suicidal Ideation

The prevalence of suicidal ideation in our sample in the 30 days prior to completing the questionnaire was 7.4% (n = 66). Table 4 shows the prevalence of this outcome in association with the study variables of interest and unadjusted and adjusted prevalence ratios.

**Table 4:** Prevalence and unadjusted and adjusted associations between suicidal ideation and studied covariates estimated using Poisson regression models. Data are prevalence ratios (PR) and corresponding 95% confidence intervals (CIs) (n = 890) (Pelotas-RS, 2020).

	n	%	Crude PR (95% CI)	Adjusted^a^ PR (95% CI)	Adjusted^b^ PR (95% CI)
Gender					
Female	755	7.55	1	1	1
Male	135	6.67	0.88 (0.44–1.74)	1.11 (0.53–2.34)	1.55 (0.71–3.37)
Ethnicity					
White	665	7.4	1	1	1
Brown	122	6.6	0.88 (0.43–1.83)	1.06 (0.50–2.26)	0.92 (0.44–1.90)
Black	103	8.7	1.18 (0.60–2.34)	1.39 (0.71–2.69)	1.46 (0.77–2.79)
Age					
Up to 30	117	8.5	1	1	1
31 to 40	365	6.8	0.80 (0.39–1.61)	0.94 (0.42–2.11)	0.83 (0.38–1.83)
41 to 50	292	7.5	0.88 (0.43–1.80)	0.97 (0.42–2.21)	0.93 (0.43–2.03)
≥ 51	116	7.8	0.90 (0.38–2.15)	1.25 (0.48–3.23)	1.45 (0.48–4.37)
Education level					
High School	330	8.5	1	1	1
Undergraduate	212	8.0	0.94 (0.53–1.68)	1.04 (0.55–1.94)	1.03 (0.56–1.92)
Graduate	348	6.0	0.71 (0.41–1.22)	1.01 (0.53–1.94)	1.19 (0.61–2.29)
Per capita income					
Up to 1 minimum wage	205	11.7	1	1	1
Up to 2 minimum wages	305	6.6	0.56 (0.31–0.98)	0.54 (0.30–0.98)	0.58 (0.33–1.01)
Up to 3 minimum wages	132	5.3	0.45 (0.20–1.02)	0.44 (0.19–1.00)	0.49 (0.21–1.14)
> 3 minimum wages	168	3.6	0.30 (0.12–0.72)^c^	0.29 (0.12–0.69)^c^	0.28 (0.11–0.68)^c^
Type of service					
Primary Care	118	8.5	1	1	1
Outpatient	92	4.3	0.51 (0.16–1.58)	0.47 (0.12–1.80)	0.49 (0.12–2.01)
Emergency	84	8.3	0.98 (0.38–2.48)	1.09 (0.37–3.23)	0.78 (0.24–2.45)
Hospital	577	7.8	0.92 (0.47–1.77)	1.07 (0.50–2.26)	0.75 (0.32–1.79)
Administrative	19	–	–	–	–
Length of service in the nursing field					
Up to 5 years	175	5.7	1	1	1
Up to 10 years	221	9.9	1.74 (0.84–3.58)	1.88 (0.85–4.13)	1.35 (0.56–3.24)
Up to 15 years	217	7.8	1.37 (0.64–2.91)	1.76 (0.78–3.97)	1.50 (0.65–3.47)
Up to 20 years	139	7.2	1.25 (0.53–2.94)	1.54 (0.62–3.48)	1.19 (0.45–3.11)
≥ 20 years	138	5.1	0.88 (0.34–2.27)	0.81 (0.28–2.33)	0.83 (0.28–2.43)
Nursing category					
Registered Nurse	319	6.0	1	1	1
Nurse technician	501	8.2	1.37 (0.81–2.32)	0.96 (0.51–1.83)	0.80 (0.40–1.56)
Nursing assistant	70	8.6	1.43 (0.59–3.47)	1.09 (0.38–3.10)	2.53 (0.80–7.97)
Length of service at institution					
Up to 5 years	520	7.1	1	1	1
Up to 10 years	160	11.2	1.58 (0.92–2.69)	1.45 (0.81–2.59)	1.10 (0.61–1.98)
Up to 15 years	71	2.8	0.39 (0.09–1.60)	0.37 (0.09–1.53)	0.38 (0.10–1.49)
≥ 15 years	134	5.2	0.73 (0.33–1.61)	0.57 (0.22–1.47)	0.72 (0.30–1.68)
Workload					
Up to 30h	194	6.7	1	1	1
Up to 36h	473	8.7	1.29 (0.70–2.36)	1.43 (0.72–2.86)	1.05 (0.57–1.95)
36h+	223	5.4	0.80 (0.37–1.71)	0.72 (0.30–1.76)	0.57 (0.25–1.27)
Second job					
No	645	8.2	1	1	1
Yes	245	5.3	0.64 (0.35–1.16)	0.61 (0.31–1.21)	0.60 (0.32–1.13)
COVID-19-specific training					
No	319	7.2	1	1	1
Yes	571	7.5	1.04 (0.64–1.70)	1.01 (0.60–1.71)	0.93 (0.55–1.56)
Evaluation of working conditions					
Good	325	5.5	1	1	1
Regular	409	6.6	1.19 (0.66–2.12)	1.27 (0.68–2.36)	1.22 (0.66–2.26)
Poor	156	13.5	2.43 (1.33–4.42)^c^	2.16 (1.13–4.13)^c^	1.53 (0.77–3.03)
Evaluation of support at work					
Good	263	6.1	1	1	1
Regular	363	6.1	0.99 (0.53–1.86)	1.10 (0.58–2.11)	0.92 (0.35–2.42)
Poor	264	10.6	1.74 (0.96–3.14)	1.58 (0.81–3.05)	0.86 (0.29–2.54)
Burden					
Light	343	6.7	1	1	1
Moderate	262	5.0	0.73 (0.38–1.43)	0.96 (0.48–1.92)	0.71 (0.35–1.40)
Heavy	285	10.5	1.56 (0.93–2.64)	1.93 (1.08–3.43)^c^	0.98 (0.47–2.05)
Current burden compared to that pre-COVID-19					
Same or decreased	337	5.6	1	1	1
Increased	553	8.5	1.50 (0.90–2.52)	2.03 (1.13–3.64)^c^	1.61 (0.88–2.96)
Involvement with COVID-19 cases					
None	288	5.9	1	1	1
Indirect work (e.g., administrative)	65	9.2	1.56 (0.64–3.81)	1.84 (0.70–4.86)	1.85 (0.72–4.71)
Contact with suspect cases	310	8.1	1.36 (0.75–2.47)	1.38 (0.74–2.56)	1.20 (0.63–2.25)
Contact with confirmed cases	277	7.9	1.34 (0.70–2.54)	1.36 (0.69–2.71)	1.25 (0.60–2.61)
COVID-19 workload proportion					
None	288	5.9	1	1	1
Up to one third	169	6.5	1.10 (0.52–2.29)	0.96 (0.43–2.17)	0.91 (0.37–2.20)
Up to two thirds	166	9.6	1.63 (0.84–3.14)	1.76 (0.90–3.44)	1.66 (0.86–3.21)
> two thirds	267	8.2	1.39 (0.75–2.57)	1.48 (0.77–2.86)	1.19 (0.60–2.33)
Lack of Personal Protective Equipment					
No	508	6.5	1	1	1
Yes	382	8.6	1.32 (0.83–2.11)	1.10 (0.67–1.80)	0.76 (0.45–1.28)
Suspected COVID-19 infection					
No	574	7.0	1	1	1
Yes	316	8.2	1.18 (0.73–1.89)	1.42 (0.84–2.37)	0.96 (0.55–1.65)
Absence from work due to suspected infection					
No	746	7.6	1	1	1
Yes	144	6.2	0.81 (0.41–1.61)	0.88 (0.43–1.80)	0.65 (0.30–1.44)
Family or close friend with COVID-19					
No	616	6.5	1	1	1
Yes	274	9.5	1.46 (0.91–2.34)	1.58 (0.94–2.65)	1.42 (0.84–2.41)
Degree of social distancing					
Light	227	8.8	1	1	1
Moderate	568	7.6	0.85 (0.51–1.42)	0.99 (0.56–1.74)	1.03 (0.58–1.81)
Intense	95	3.2	0.35 (0.10–1.17)	0.42 (0.13–1.38)	0.53 (0.15–1.76)
Risk group					
No	606	6.1	1	1	1
Yes	284	10.2	1.67 (1.05–2.66)^c^	1.60 (0.96–2.65)	1.37 (0.81–2.31)
Problems with/abuse of alcohol					
No	824	6.8	1	1	1
Yes	66	15.1	2.22 (1.19–4.16)^c^	2.56 (1.31–4.96)^c^	1.92 (0.98–3.78)
Tobacco use					
No	748	6.8	1	1	1
Yes	142	10.6	1.54 (0.89–2.67)	1.42 (0.79–2.54)	1.09 (0.62–1.92)
Current use of psychotropic drugs					
No	703	4.8	1	1	1
Yes	187	17.1	3.53 (2.24–5.57)^d^	3.51 (2.14–5.76)^d^	3.14 (1.87–5.26)^d^

**TOTAL**	**880**	**7.4**			

^a^ Adjusted for: gender; age; and per capita income.

^b^ Adjusted for: per capita income; length of service at the institution; workload; second job; work conditions evaluation; burden comparison (post-COVID-19); COVID-19 case on family or close friend; alcohol problems; and current use of psychotropic drugs.

^c^ p-value < 0.05

^d^ p-value < 0.001

In the model where the adjustment was performed for all potential confounders related to the outcome, suicidal ideation showed a strong positive association with psychotropic drug use (PR: 3.14; 95% CI: 1.87–5.26), but this outcome was inversely correlated with having a per capita income greater than three minimum monthly wages (PR: 0.28; 95% CI: 0.11–0.68). When only adjusted for gender, age, and per capita income, suicidal ideation was also associated with assessing one’s working conditions as poor (PR: 2.16; 96% CI: 1.13–4.13), reporting a heavy burden at work (PR: 1.93; 95% CI: 1.08–3.43), reporting increased burden post-pandemic (PR: 2.03; 95% CI: 1.13–3.64), and problems with alcohol (PR: 2.56; 95% CI: 1.31–4.96).

## DISCUSSION

The COVID-19 pandemic has had substantial negative effects on the mental health of many healthcare professionals. This study aimed to identify factors associated with mental health outcomes to develop strategies for mitigation. Importantly, our study design included a well-defined sample frame and strict recruitment protocol, thus meeting recommendations emerging from the field^12^ .

The results notably indicated a high prevalence of major depressive episodes (36.6%) and MPDs (43.9%) in our sample, thus pointing to the need for interventions to promote mental health among nursing professionals.

However, the instruments used to track these outcomes vary among studies. For example, some authors have used the Patient Health Questionnaire-9 (PHQ-9)^6^ , the Zung Self-Rating Depression Scale (SDS)^18^ , and the Hamilton Depression Scale (HAMD)^19^ (among others) to screen for depression among healthcare professionals since the pandemic began.

The prevalence of depression found in the current study was higher than that reported among other studies using the PHQ-9 [(12.2%)^20^ and (13.5%)^9^ ], but lower than the results observed by one study (50.4%)^6^ . However, our results were similar to the pooled prevalence calculated in a meta-analysis which included the above three studies: 36.7% (95% CI: 7.7–69.2, I^2^ = 100%)^7^ .

The results reported herein suggest that pre- and post-pandemic depression prevalence is significantly higher among nursing professionals than in the general population. In a population-based study conducted before the pandemic in the same municipality (Pelotas), the prevalence of depression was 19.0% (CI: 15.4–22.7)^14^ . In a cross-sectional online survey conducted among the general population in China, a prevalence of 20.1% (CI: 19.2–21.0) was reported^5^ .

During the pre-pandemic period in Brazil, studies reported depression prevalence of 21.3% and 27%, respectively^21 , 22^ . This suggests a potentially greater occurrence of depressive episodes among nursing professionals during the pandemic. However, few studies to date have compared results obtained from the same sample of nurses both before and during the pandemic.

Our results similarly suggest a greater occurrence of MPDs among the nursing professionals in our sample than both the general population and nursing professionals working in other countries (both pre and post-pandemic). Our results are similar to those found among Pakistani doctors, in whom a MPD prevalence of 42.7% was found using the SRQ-20^23^ .

Two studies conducted in the pre-pandemic period among Brazilian nurses suggested the prevalence of MPD was 33.3%^24^ and 35%^25^ . We emphasize again the methodological variability among studies tracking these mental health outcomes both before and during the pandemic period.

We found a suicidal ideation prevalence of 7.4% in our sample. This is higher than that found for 12 months in a population-based study in Brazil conducted in 2003 but lower than that found for 30 days among the general population of the United States of America (10.7%) during the COVID-19 pandemic^26^ .

Nursing professionals compose approximately half of the world’s health workforce^2^ . Their work involves several challenges, including potential ethical dilemmas, working with human suffering, long hours, low pay, lack of time and appropriate space to rest, burden, lack of resources, and low appreciation by other team members. These factors have previously been recognized to cause worsening in the nurses’ mental health^24 , 25 , 27^ , and their effects may be exacerbated by the pandemic.

Thus, we emphasize the associations found for both major depressive episodes and MPDs with burden, a poor assessment of the support received by one’s service, and suspected COVID-19 infection among our results. The association observed for major depressive episodes and lack of PPE is also noteworthy. These results are consistent with previous studies that investigated the repercussions of the pandemic in nurses from other countries.

Positive associations between depression and/or anxiety and suspected infection with COVID-19 have been reported in studies conducted in China^28^ and Iran^20 , 29^ . In turn, the perception of support received by the service was negatively associated with poor self-rated health in the study^28^ , in addition to being seen as one of the greatest needs for reducing the psychological burden in a study conducted among German nurses^30^ . Finally, studies conducted among healthcare professionals in Italy^31^ , Iran^20 , 29^ , and Portugal^32^ observed that adequate PPE provision was an important predictor of better psychological outcomes.

Although the results in our sample suggest a higher prevalence of depression and MPD in comparison to the general population or other pre-pandemic samples of nurses, few pandemic-specific study variables were related to these outcomes in our study. However, we can suggest that common challenges faced by the nursing teams were exacerbated by the pandemic.

A note of caution is also necessary regarding interpretations of depression and MPD prevalence. Importantly, screening for these outcomes using instruments, even validated ones, does not confirm a diagnosis, and many responses to questions in these instruments may reflect normal adaptive responses to a stressful period. Therefore, it is not necessary to pathologize conditions that could be treated with the adoption of simple measures, such as improving professional support and working conditions.

Some hospitals in China implemented psychological assistance services in response to large numbers of workers screening positive for adverse mental health outcomes. Interestingly, workers were reluctant to participate in the interventions offered^4^ . Through interviews with 13 medical teams at Xiangya Hospital, a study found that many workers were more immediately concerned on biosafety and lack of knowledge on COVID-19 among the reasons for refusing mental healthcare. Rather than mental health assistance, workers reported needing more uninterrupted rest and sufficient PPE to perform their duties.

Finally, readers should consider the limitations of the current study. First, our study was cross-sectional, therefore reverse causality cannot be ruled out. We must also consider the risk of response bias given that the research involved self-reporting by the participants.

We chose to exclude nursing professionals who were absent from work during the data collection period from our sample. This exclusion criterion must also be considered a limitation because it may be the case that such individuals were absent from work as a result of mental health issues related to the pandemic. Moreover, nursing professionals with mental healthcare needs may have been more likely among those eligible to participate in this study.

Although associations observed among our outcomes of interest and participant income are plausible and in line with prior study findings, one should consider that there were 80 missing observations for the income variable in our dataset. A lack of reference values for the same population in the pre-pandemic period represents another limitation. This makes the comparison and interpretation of results more difficult, highlighting that longitudinal studies are urgently needed.

We observed a limited number of observations for suicidal ideation. This outcome demonstrated only weak associations with several study variables (i.e., evaluation of support at work, current burden, and burden pre-pandemic comparison) after adjustment for confounders. A larger sample may be required to detect stronger associations of suicidal ideation with other variables of interest.

Finally, it is necessary to point out that the selection of variables to compose the model with purely statistical criteria has been criticized by epidemiology theorists^33^ . However, we understand that the selection of confounders through a selection based on statistical criteria helped us to enrich the analysis through identifying and including new variables in the literature (those related to the context of the COVID-19 pandemic), for the which relationships were not yet well defined.

## CONCLUSIONS

Our results point to a high prevalence of major depressive episodes and MPDs among the nursing professionals studied. Associations observed for these outcomes included suspected COVID-19 infection, burden at work, a rating of support received by one’s service as poor, and a lack of PPE. Our study, therefore, suggests that factors related to services are associated with the mental health status of nursing professionals. There is a need to improve working conditions, especially by ensuring adequate dimensioning to avoid the burden. Employers should provide their employees with psychological and social support and implement adequate biosafety measures. Such measures are arguably needed to promote feelings of security and to reduce anxiety linked to pandemic-related uncertainties and risk of infection.
